# Gold-Induced Cytokine (GOLDIC) for the Management of Knee Osteoarthritis: A Systematic Review

**DOI:** 10.7759/cureus.73040

**Published:** 2024-11-05

**Authors:** Adarsh Aratikatla, Vibhu Krishnan Viswanathan, Samir Ghandour, Vijay Kumar Jain, Ashim Gupta

**Affiliations:** 1 Medical School, The Royal College of Surgeons in Ireland, Dublin, IRL; 2 Department of Orthopedics, Dr Rela Institute and Medical Center, Chennai, IND; 3 Foot and Ankle Research and Innovation Laboratory, Harvard Medical School, Boston, USA; 4 Department of Orthopedics, Atal Bihari Vajpayee Institute of Medical Sciences, Dr Ram Manohar Lohia Hospital, New Delhi, IND; 5 Department of Regenerative Medicine, Future Biologics, Lawrenceville, USA

**Keywords:** autologous conditioned serum, autologous peripheral blood-derived orthobiologics, goldic, gold-induced cytokine, knee osteoarthritis, orthobiologics, regenerative medicine

## Abstract

Gold-induced cytokine (GOLDIC) is a novel orthobiologic approach utilizing gold particles to produce a serum rich in immunoregulating cytokines and growth factors, which is being explored for its potential in tissue regeneration and treating musculoskeletal issues like knee osteoarthritis (OA). This study aims to review its mechanism of action along with the outcomes of *in vitro*, preclinical, and clinical studies, with a secondary focus on documenting clinical trials related to its use in OA of the knee. A systematic search was conducted in four databases (Embase, Scopus, PubMed, Web of Science) for studies on GOLDIC therapy for knee OA, using specific keywords related to knee anatomy and OA. *In vitro* studies demonstrated that gold-containing compounds reduce nitric oxide production in chondrocytes, mitigating catabolic processes. Pre-clinical trials in horses with lameness showed significant symptom improvement. Clinical studies reported substantial improvements in pain, function, and joint homeostasis, with reduced synovial effusion and cytokine modulation following GOLDIC therapy. GOLDIC therapy, in addition to orthopedic indications such as for the management of OA of the knee, has also been investigated in non-orthopedic settings with early promising results. However, more research is needed to fully understand its mechanism of action and establish its clinical utility.

## Introduction and background

Historical background

Over the last two decades, the possibility of regenerating the dead, diseased, and degenerated tissues along with recovering their innate biomechanical properties has been extensively researched [1]. Such innovative research in the field of “Tissue Engineering and Regenerative Medicine (TERM)" has found diverse applications across various subspecialties of health care in the last few decades [2]. The advancements in TERM have been so immense that the governing or regulatory bodies across the world have put forth guidelines and ethical recommendations for the production, storage, and administration of different cellular and acellular biological agents [1,3].

The concept of gold-induced cytokine (GOLDIC) therapy was first introduced by the German physicist, Ulrich Schneider in the year 2014 [4]. Although gold had historically been utilized in the management of rheumatoid arthritis, its use was abandoned in view of serious systemic adverse effects [5]. The phenomenon of proliferation of anti-inflammatory cytokines through prolonged incubation of whole blood in tubes coated with hydrophilic gold particles, which, in turn, leads to the formation of serum with strong anti-inflammatory effects, forms the basis for its therapeutic capability to instigate regenerative processes within diverse tissues [1,4,6-8]. This therapy was invented and introduced by Arthrogen GmbH (Germany) [1,9].

Introduction

Platelets and their derivatives, various cytokines, and growth factors have been acknowledged to play a crucial role in the realm of tissue regenerative medicine [10]. Among the diverse orthobiologic agents, “GOLDIC’ (Arthrogen GmbH, Gmund am Tegernsee, Germany) has recently gained massive interest among researchers and global experts [1,4,6-8,11-13]. GOLDIC is a novel, innovative approach in the field of regenerative orthopedics, which utilizes specialized particles of gold to produce a conditioned serum rich in immunoregulating cytokines [8]. To prepare such orthobiologic agents for administration prior to injection, patients’ blood samples are collected and prepared with the help of GOLDIC tubes, which include specially designed gold particles and a special filter capable of preventing the particles, platelets, and cells from being injected. During the approximate 24-hour-long incubation process, gold particles behave as catalysts for monocytes in the production of autologous cytokines. These tubes are then centrifuged to separate the serum from the remaining blood components. The produced serum is highly bioactive and rich in growth factors (GFs) and cytokines, which can be injected directly into the tissues or bloodstream [1]. 

In view of its anti-inflammatory properties, GOLDIC’s utility in the context of tissue regeneration has been growingly explored. GOLDIC has been proposed as a treatment strategy in the management of different musculoskeletal problems, such as tendinopathies (i.e. lateral epicondylitis, Achilles tendinopathy, plantar fasciitis), osteoarthritis (OA), and lumbar canal stenosis [3,4,6,9,11]. Knee OA is a common musculoskeletal pathology for which diverse orthobiologic agents have already been trialed during different stages of the disease [14]. Recently, the role of GOLDIC therapy has also been explored in the management of the early stages of knee OA, and certain reports have demonstrated a considerable ability of GOLDIC therapy to effectuate cartilage regeneration [7-9].

The primary objective of this study is to review the mechanism of action focusing on the intra-articular (IA) mechanical, biological, and chemical homeostatic properties, along with documenting the *in vitro*, pre-clinical, and clinical outcomes of GOLDIC therapy for the treatment of knee OA. The secondary objective is to document the ongoing clinical trials registered on different trial protocol repositories related to GOLDIC for the management of knee OA.

## Review

Methods

Search Criteria

**Figure 1:**
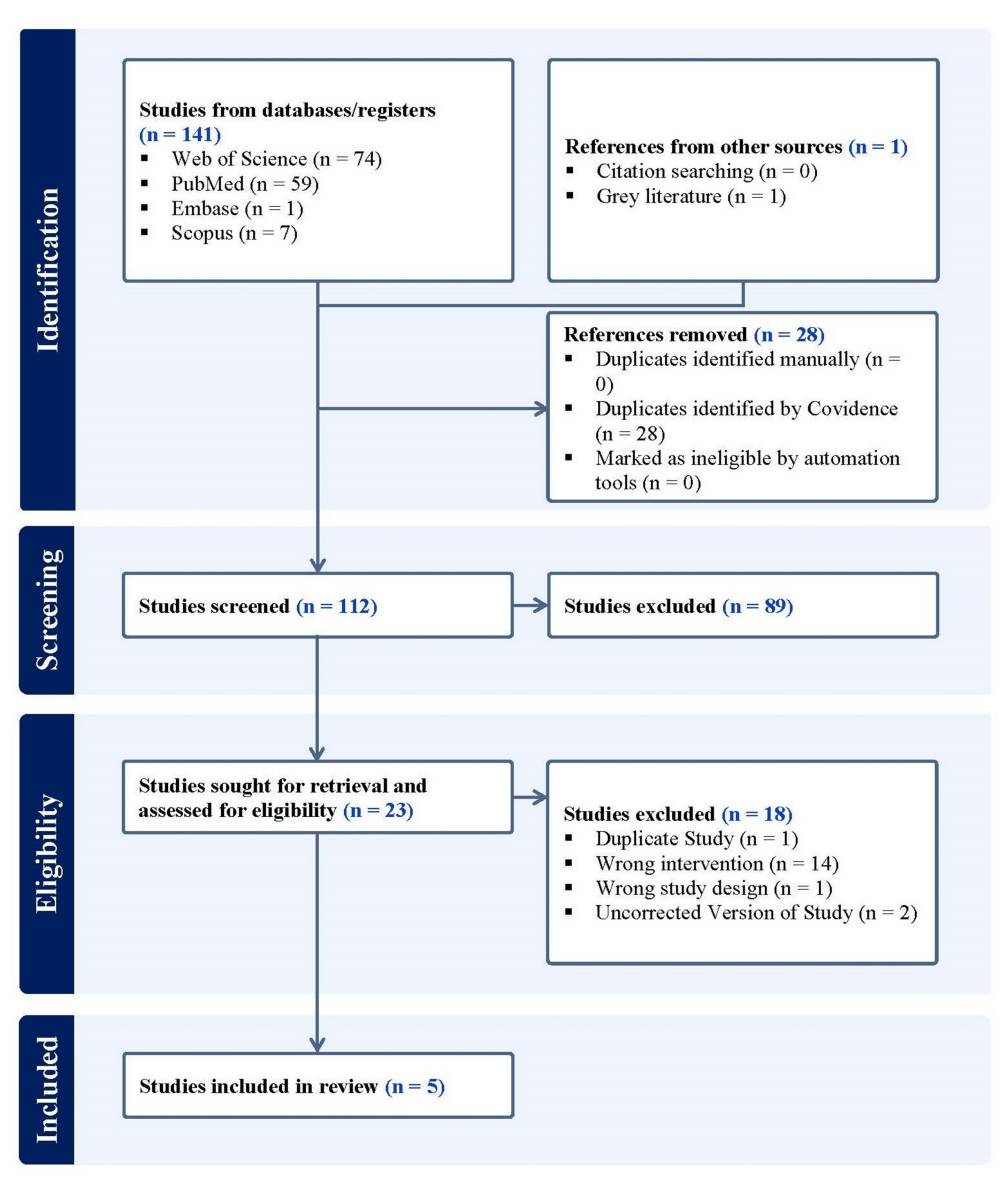
A PRISMA flow diagram outlining the record identification and selection process. PRISMA: Preferred Reporting Items for Systematic Reviews and Meta-Analysis

A search was done using the keyword terms, (‘GOLDIC’ OR ‘gold-induced cytokines’ OR ‘gold induced cytokines’ OR ‘gold induced’ OR ‘gold-induced’ OR ‘gold cytokines’) AND ('knee' OR ‘femur’ OR ‘femoral’ OR ‘tibia’ OR ‘tibial’ OR ‘patella’ OR ‘patellar’ OR ‘cruciate’ OR ‘collateral’ OR ‘fibula’ OR ‘fibular’ OR ‘tibiofemoral’ OR ‘femorotibial’ OR ‘patellofemoral’ OR ‘tibiofibular’ OR ‘knee capsule’) AND ('osteoarthritis' OR 'arthritis' OR ‘degenerative joint’ OR ‘osteoarthrosis’ OR ‘degenerative arthritis’ OR ‘wear and tear arthritis’) in four online journal databases, including Embase, Scopus, PubMed, and Web of Science, for articles published in English up to October 10, 2024, while following the Preferred Reporting Items for Systematic Reviews and Meta-Analysis (PRISMA) guidelines [15]. All studies using GOLDIC as an intervention for the treatment of knee OA were accepted, including *in vitro*, preclinical, and clinical studies. Studies not utilizing GOLDIC alone or not exploring its use for the management of knee OA were excluded (Figure 1). In addition, we searched Clinical Trials Registry - India (CTRI), ClinicalTrials.gov, and Chinese Clinical Trial Register (ChiCTR) using the aforesaid search terms to find listed ongoing clinical trials on the use of GOLDIC for the management of knee OA.

Results

There are one, one and three in vitro, pre-clinical and clinical studies, respectively, that assessed GOLDIC in the context of knee OA (Table 1).

**Table 1:** Summary of main findings of included in vitro studies involving gold-induced cytokine (GOLDIC) for the management of knee disorders. DMARDs: Disease-modifying anti-rheumatic agents; VAS: Visual Analogue Score; WOMAC: Western Ontario and McMaster Universities Osteoarthritis Index; iNOS: inducible NO synthase

Author [Reference]	Study Type	Sample Size	Main Findings
Vuolteennaho et al. [11]	In vitro	Not applicable	Aurothiomalate and hydroxychloroquine have been identified as effective DMARDs that inhibit IL-1β-induced NO production in chondrocyte cultures and human osteoarthritic cartilage. Methotrexate and leflunomide have shown limited effects in this context. Nitric oxide is implicated in the pathogenesis of OA and RA, as it inhibits collagen and proteoglycan synthesis, induces chondrocyte apoptosis, and promotes the activation of metalloproteinases, thereby contributing to cartilage destruction. The mechanism by which these DMARDs exert their effects involves the suppression of iNOS expression and the inhibition of NF-kB activation, which is crucial for iNOS transcription, thereby reducing the inflammatory and destructive cytokine environment in chondrocytes.
Schneider et al. [4]	Pre-clinical	37	Improved lameness scores and reduced swelling/effusion were noted
Tulpule et al. [7]	Clinical (observational study)	106	VAS (pain) scores significantly decreased from 6.31 pre-injection to 0.43 at 1 year (p < 0.001 at all intervals). WOMAC (function) scores significantly improved from 20.12 pre-injection to 76.27 at one year (p < 0.001 at all intervals).
Schneider et al. [8]	Clinical (Phase 2a, proof-of-concept, open label)	89	There was a significant reduction in VAS scores over time, with a mean pre-injection score of 6.31, which reduced to 0.43 at one year post-injection. WOMAC scores improved over time, with a mean score of 20.12 pre-injection, which increased to 76.27 at one year, indicating significant improvement in function.
Pithadia et al. [16]	Clinical (case report)	1	VAS pain score improved from 9/10 to 1. WOMAC scores significantly improved, showing no functional impairment by 3 months. Weight reduced from 105 kg to 95 kg over nine months, no adverse events noted.

In Vitro Studies

Vuolteennaho et al. investigated the effects of disease-modifying anti-rheumatic agents (DMARDs) on nitric oxide (NO) production in both murine chondrocytes and human osteoarthritic cartilage cell lines. Regarding the murine chondrocyte cell line H4, the authors determined that aurothiomalate, a gold-containing biologic, along with other DMARDs including, hydroxychloroquine, methotrexate, and leflunomide, curtailed interleukin-1-beta (IL-1β)-induced nuclear factor kappa-light-chain-enhancer of activated B cells (NF-kB) production, therefore mitigating subsequent signaling of inducible NO synthase (iNOS) [11]. Furthermore, in the human cell lines, aurothiomalate was shown to decrease IL-1β-induced NO production significantly more than other DMARDs, i.e. methotrexate and leflunomide [11].

Pre-clinical Studies

Schneider et al. assessed the effects of GOLDIC on varying lameness-associated equine pathologies [4]. In this study, 37 horses with clinical lameness (chondromalacia: n=19, soft tissue disorders: n=18) were treated with four injections of GOLDIC therapy. There was a significant reduction of lameness, joint effusion, and swelling in all cases within three weeks of therapy (p<0.05). At three and six months, all horses included in the study were asymptomatic.

Clinical Studies

Tulpule et al. evaluated the safety and functional outcome following GOLDIC serum injection in patients with Kellgren-Lawrence (KL) grades 3 or 4 knee OA [7]. A total of 106 knees (n=65 patients) in this multi-center study were assessed using the visual analogue score (VAS) and Western Ontario and McMaster Universities Osteoarthritis Index (WOMAC) scores at baseline and 4, 12, 24, and 52 weeks of follow-up. A total of four doses of 4 ml of ultrasound-guided IA GOLDIC serum injections were administered at three to six-day intervals. The results showed a statistically significant improvement in pain and functional outcome at all the postoperative follow-up time points following the therapy (although there was treatment failure in three patients at the end of one year). 

Schneider et al. evaluated 64 patients (n=89 knees, mean age: 64.8 years) undergoing IA GOLDIC therapy (four doses of ultrasound-guided IA injections every third to sixth day) for moderate to severe knee OA [8]. In their series, the authors reported significant improvement in functional outcome scores, WOMAC and knee injury and osteoarthritis outcome score (KOOS), at all follow-up time points, with minimal clinically important differences (MCIDs) observed at all time points for all KOOS subscales. In addition, in this series, there were increased levels of IA gelsolin following the GOLDIC therapy (even after the first injection). Interestingly, the raised gelsolin level was accompanied by reduced synovial fluid production. Based on these findings, the authors reported that GOLDIC therapy affects overall joint homeostasis, reduces synovial effusion, modulates cytokine levels, and improves functional outcomes. In this series, the failure rate (defined as the need to undergo TKA) was seen in nine (14.1%) patients with a mean time to failure of around 32 months. In the same series, 94% of joint effusions were also eliminated after the fourth dose of GOLDIC therapy, while 67% of effusions were reduced after the third injection [8].

Pithadia et al. presented the case of a 60-year-old female patient with bilateral, grade 4 knee OA. The VAS pain score improved from 9/10 to 1/10. Initial WOMAC scores showed significant functional impairment (i.e. right knee=76, left knee=81). Twelve months post-IA GOLDIC injections, WOMAC scores improved to 17 in the right knee and 23 in the left knee. Both scores showed rapid improvement, with no functional impairment observed at three months. No significant adverse events were reported during follow-up [16].

On-going Clinical Studies

As of October 10, 2024, no clinical trials were listed on ClinicalTrials.gov, CTRI, or ChiCTR to evaluate the safety and/or effectiveness of GOLDIC to manage OA of the knee.

Discussion

Due to the increasing longevity of the global population, the prevalence of OA in the knee joints has been progressively on the rise over the past decades [17]. Total knee arthroplasty (TKA) remains the final option for the definitive management of symptomatic, KL grades 3 or 4 OA knees [18]. However, patients with KL grades 2 or 3 OA knees also suffer substantially due to chronic pain and associated functional limitations and impairment [19]. In these situations, less invasive options including IA injections, have been acknowledged as excellent alternatives [20]. Among such IA injection options, hyaluronic acid and corticosteroid injections have been the most widely used substances, albeit with significantly shorter duration of pain relief and minimal influence over the natural history of disease progression [21,22]. On the other hand, there is a rising trend in the utilization of biological agents such as mesenchymal stem cells, autologous peripheral blood-derived orthobiologics, bone marrow aspirate concentrate, and exosomes in the field of regenerative orthopedics [23-33]. The long-term efficacy of these substances and their relative roles in the management of OA of different KL grades are still largely unclear [8]. 

Mechanism of Action of GOLDIC

The modality by which GOLDIC exerts its therapeutic effects is still widely unknown; however, there are several theories regarding its mechanism of action [7,8]. It has been proposed that hydrophilic gold particles trigger a series of molecular cascades, which culminate in the development of a serum rich in diverse GFs and cytokines. There is evidence that gold-based regenerative therapy results in the downregulation of CD4+ T-cells, B-cells, and proinflammatory cytokines such as interleukin-9 (IL-9) and tumor necrosis factor (TNF-α) in patients’ serum, which have all been described as crucial mechanisms for the anti-inflammatory and pro-regenerative properties of the gold molecule [1,34,35]. Some *in vitro* studies show that one of the purported mechanisms of action of this technology is the increase in the plasma and synovial gelsolin levels following GOLDIC injections [7,8]. Gelsolin is an actin-binding protein that exists as a component within the cellular cytoskeleton and blood plasma [13,36-38]. It is responsible for the regulation of diverse cellular functions such as the maintenance of cellular viscoelasticity, phagocytosis, apoptosis, thrombocyte activation, and cellular motility [8]. Studies have demonstrated the ability of plasma gelsolin to behave as a buffer and impede the progression of inflammatory activities [39]. For example, reduced levels of gelsolin in the plasma have been reported in sepsis and inflammatory arthropathies, and decreased concentrations within the synovial tissues have been associated with rheumatoid arthritis and degenerative pathologies of the joints [4,39]. In the first human trial on patients with Achilles tendon injury, GOLDIC injection was associated with substantial upregulation of plasma gelsolin and granulocyte colony-stimulating factor (G-CSF) levels, both of which were attributed to the clinical and radiological TA regeneration [6]. In addition, GOLDIC therapy may also act through the enhancement of diverse pro-anabolic and anti-catabolic parameters [6,8,40].

Although many studies have presented promising early evidence regarding the efficacy of GOLDIC therapy in diverse degenerative diseases through animal studies, the recommendation regarding its implementation in routine clinical practice is still unclear.

GOLDIC Therapy for the Treatment of Knee OA

The assessment of GOLDIC therapy for knee OA reveals significant similarities in outcomes across various studies, underscoring its potential as a viable treatment option. In a clinical study by Schneider et al., the efficacy of GOLDIC was evaluated through standardized measures such as the WOMAC, which demonstrated consistent improvements in pain and functional scores among patients receiving IA injections of GOLDIC® [8] This aligns with findings from Pithadia et al., who noted that GOLDIC therapy positively influenced patient outcomes, particularly in those with advanced stages of knee OA, highlighting its regenerative properties [16].

Moreover, these studies emphasize the long-term benefits of GOLDIC therapy compared to traditional treatments like platelet-rich plasma and hyaluronic acid, which often yield inconsistent results and are limited to short-term relief. For instance, Tulpule's observational study indicated that GOLDIC therapy resulted in sustained improvements in functional scores for up to four years, a stark contrast to the temporary benefits associated with other biologic therapies [7]. This consistency in outcomes across different studies suggests that GOLDIC therapy may offer a more reliable and effective approach to managing Knee OA, particularly in patients who have not responded well to conventional treatments. The standardization of the GOLDIC preparation process further enhances its reliability, ensuring that patients receive a consistent therapeutic product [16]. Overall, the convergence of positive outcomes across multiple studies reinforces the potential of GOLDIC therapy as a promising treatment modality for knee OA.

GOLDIC Therapy in Other Musculoskeletal Pathologies

In addition to OA of the knee, the use of GOLDIC has been investigated in other musculoskeletal pathologies. For example, in a recent prospective, randomized controlled trial, Godek et al. demonstrated that epidural GOLDIC serum injections demonstrated the highest mean differences and number of patients with a MCID for the primary outcome measures using the numerical rating scale (NRS), Oswestry disability index (ODI), Zurich claudication questionnaire and EuroQol 5 Dimensions 5 Level (EQ-5D-5L) questionnaire among the three strategies evaluated (epidural GOLDIC therapy vs. epidural steroid injection vs. manual therapy) [3]. The study confirmed the safety of the therapy and attributed the benefits of epidural GOLDIC serum injections to anti-edema effects and mitigation of venous stasis. 

In a prospective clinical series of patients with Achilles tendinopathy, four peritendinous injections of GOLDIC serum resulted in a statistically significant improvement in VAS scores (at 4, 12, 24, and 52 month-follow-up time points) and complete regeneration of the Achilles tendon on magnetic resonance imaging (MRI) at the one-year follow-up time point. There were no serious adverse events related to GOLDIC serum in this series [4,6].

Melo et al. demonstrated the safety and efficacy (significant improvement in VAS, foot and ankle disability index (FADI), global rating change (GroC) scores) of GOLDIC serum injection (four doses) in an Olympic equestrian suffering from recalcitrant plantar fasciosis [41]. The patient could return to active, high-level sports within eight weeks of therapy without any limitations. 

Potential Benefits of GOLDIC Therapy in Non-orthopedic Settings

In addition to musculoskeletal pathologies, GOLDIC has been investigated in other medical specialities; further research of GOLDIC in these fields may provide insight as to what the mechanism of action of this modality may be. Further research is warranted in both orthopedic and non-orthopedic settings to definitively assess the mechanism of action of GOLDIC [1,7].

The potential regenerative benefits of GOLDIC may help facilitate cardiac tissue regeneration after myocardial infarction, promoting angiogenesis, and enhancing the survival of cardiomyocytes; however, a study conducted by Cordes et al. has demonstrated that GOLDIC interventions may impair ventricular remodeling after a myocardial infarction [1,42]. Additionally, there is a substantial potential for this technology to stimulate neuronal growth, mitigate inflammatory processes, preserve neural function, and facilitate regeneration. These properties can be utilized to treat diverse neurodegenerative and autoimmune, inflammatory neurological diseases such as Guillain-Barre syndrome [1]. Furthermore, GOLDIC therapy can potentially augment wound healing in chronic diabetic ulcers and non-healing wounds. The ability of this treatment to rejuvenate the skin as well as enhance tissue remodeling and cell proliferation can potentially find applications in diverse cosmetic and aesthetic procedures [1]. Moreover, the anti-inflammatory abilities of the GOLDIC therapy can be utilized to treat autoimmune pathologies like Crohn’s disease, psoriasis, inflammatory arthropathies, post-COVID-19 syndrome, or seasonal pollen-based allergies field [43,44]. The ability of the GOLDIC therapy to enable the regeneration of tissue can be utilized in challenging clinical scenarios such as organ transplantation, tissue engineering, or complex maxillofacial reconstructions that may require continuous systemic medical management and immune modulation [1].

A definitive conclusion cannot be made regarding the therapeutic potential of GOLDIC in the aforementioned non-orthopedic conditions, as the safety and efficacy of this intervention have not been thoroughly studied, highlighting the need for additional randomized controlled trials.

Limitations and Future Directions of GOLDIC for Knee OA

There are several major limitations of this review. There is a gross lack of literature on all types of investigations, including in vitro, preclinical, and clinical studies. More in vitro and pre-clinical studies are warranted to completely understand the mechanism of action of GOLDIC. In addition, the cohorts used in the clinical studies were not large enough to extrapolate to larger patient populations. The current body of evidence only consists of observational studies and small clinical studies, necessitating adequately powered, multi-center, blinded, randomized controlled trials with extended follow-up time periods.

## Conclusions

GOLDIC therapy presents a novel and promising approach for managing knee OA, demonstrating significant improvements in pain relief and functional outcomes as evidenced by recent studies. The mechanism of action, although poorly understood, suggests that this therapy involves the modulation of inflammatory pathways and enhancement of regenerative factors, which may support its potential as a non-invasive treatment option; the current body of evidence is limited by the small sample sizes and observational nature of the studies conducted thus far. To fully establish the efficacy and safety of GOLDIC, larger randomized controlled trials are essential. These studies should aim to explore the long-term benefits and potential adverse effects of this therapy across diverse patient populations. Ultimately, GOLDIC could become a part of a comprehensive treatment strategy for patients suffering from knee OA, particularly for those seeking alternatives to traditional pharmacological and surgical interventions.
